# Association between Gross National Income per capita and COVID-19 vaccination coverage: a global ecological study

**DOI:** 10.1186/s12889-023-17241-y

**Published:** 2023-12-04

**Authors:** Dennis Ogeto Nyachoti, Pierre Fwelo, Andrew E. Springer, Steven H. Kelder

**Affiliations:** 1 Texas Department of State Health Services, Epidemiology and Surveillance Unit, Austin, TX USA; 2 Department of Health Promotion and Behavioral Sciences, The University of Texas Health Science Center at Houston School of Public Health, El Paso, TX USA; 3 Department of Epidemiology, Human Genetics & Environmental Sciences, The University of Texas Health Science Center at Houston School of Public Health, Houston, TX USA; 4 Department of Health Promotion and Behavioral Sciences, The University of Texas Health Science Center at Houston School of Public Health, Austin, TX USA; 5 Department of Epidemiology, Human Genetics & Environmental Sciences, The University of Texas Health Science Center at Houston School of Public Health, Austin, TX USA

**Keywords:** SARS-CoV-2, Coronavirus, COVID-19, Vaccines, Vaccination, Vaccine equity, Low-income

## Abstract

**Background:**

Coronavirus 2019 (COVID-19) pandemic has claimed over six million lives and infected more than 650 million people globally. Public health agencies have deployed several strategies, including rolling out vaccination campaigns to curb the pandemic, yet a significant proportion of the global population has not received the COVID-19 vaccine. We assessed differences in COVID-19 vaccination coverage by Gross National Income (GNI) per capita of WHO members (i.e., countries, areas, and territories, *n* = 192) and by WHO member regions (*n* = 6).

**Methods:**

Using an ecological study design, we analyzed publicly available data from the WHO website merged with the World Bank’s GNI per capita data. We included a total of 192 WHO members and six WHO regions in the analysis. We utilized negative binomial regression to assess the associations between the GNI per capita and COVID-19 vaccination coverage (cumulative number of persons fully vaccinated and/or received at least one dose of the vaccine per 100 population), and ANOVA test to assess the differences in vaccination coverage per WHO regions.

**Results:**

Low GNI per capita WHO members had significantly lower full vaccination coverage (aRR 0.30, 95% CI 0.22—0.40) compared to high GNI per capita WHO members. These members were also 66% less likely to receive at least one dose of the vaccine (aRR 0.34, 0.26—0.44) relative to high GNI per capita WHO members. Africa region had a significantly lower fully vaccination coverage (aRR 0.71, 95% CI 0.36—0.54) and received at least one dose of the COVID-19 vaccine (aRR 0.78, 95% CI 0.62—0.99) than Europe region. Conversely, the Western Pacific region had significantly higher fully vaccination coverage (aRR 1.40 95% CI 1.12—1.74) and received at least one dose of COVID-19 vaccines (aRR 1.40 95% CI 1.14—1.73) relative to European region.

**Conclusion:**

WHO members with low GNI per capita and the African region reported significantly lower COVID-19 vaccination coverage than those with high GNI per capita or other regions. Efforts to strengthen and promote COVID-19 vaccination in low-income WHO countries and African region should be scaled up.

## Background

Coronavirus disease 2019 (COVID-19) is a highly contagious disease caused by the Acute Respiratory Syndrome Coronavirus-2 (SARS-CoV-2) [1–3]. Even with increased prevention and control efforts, COVID-19 continues to burden public health systems globally [4–6]. As of January 9, 2023, over 650 million cases and 6.8 million deaths had been recorded worldwide [7]. In efforts to control the pandemic, public health agencies have deployed measures, such as hand hygiene, masking, social distancing, vaccination, and treatment. Of these, vaccination remains the most viable strategy to fight against the pandemic [8]. Since its approval and launch in December 2020, SARS-CoV-2 vaccination program has been rolled out in all countries [9, 10]. Yet a significant proportion of the world’s population has not been vaccinated against COVID-19 [11, 12].

Low vaccination coverage is a precursor for emergence of COVID-19 variants and mutations [10], eventually subverting vaccination success. For instance, the WHO recently reported more transmissible Omicron virus strains than the previous subtypes [13, 14]. Increasing vaccination coverage (getting fully vaccinated against COVID-19) would help reach herd immunity and protect vulnerable populations [15]. In addition, vaccination prevents COVID-19-related deaths and severe COVID-19 infections [16]. Affordability, accessibility, and availability are major drivers for global vaccination [5, 6]. Thus, world leaders established a global coalition called COVAX [6, 17], to promote vaccine equity and distribution, yet global vaccination rates remain low [11].

Vaccine hesitancy, high illiteracy levels, poverty, and lack of political will are some of the main reasons for low vaccination coverage [18, 19]. Other studies have also indicated that the type of vaccine available could influence vaccine coverage [20, 21]. Moreover, country’s income may predict vaccine coverage, availability, and health care infrastructure [22, 23]. Despite the importance of income as a predictor for COVID-19 vaccination coverage, research is limited on the association between country-level income and region-level income and COVID-19 vaccination rates. Understanding this association will help governments, public health agencies, and world opinion leaders better plan for programs that promote COVID-19 vaccination coverage. In addressing this research gap, this study assessed the association between Gross National Income (GNI) per capita of WHO members (i.e., countries, areas, and territories) and WHO regions with COVID-19 vaccination coverage using an ecological study design.

## Methods

### Data source and study design

Using an ecological study design, we analyzed publicly available data from the WHO pooled vaccination dataset (*n* = 228 WHO members) merged with the World Bank’s GNI per capita data (*n* = 215 members). The WHO vaccination data are extracted and compiled from members’ reports and third parties [24]. The dataset is updated weekly with vaccine introduction and administration by WHO members. We used data as of June 4, 2022. A detailed description of the WHO vaccination dataset and data collection can be found elsewhere [7, 25]. The World Bank GNI per capita data is routinely collected and compiled using reports published by its member countries’ national statistical authorities, the Organization for Economic Cooperation and Development (OECD), the International Monetary Fund, or directly from countries official data. We selected 2019 GNI per capita data as an indicator of the WHO members’ economy prior to the pandemic. A detailed description of the World Bank dataset and data collection can be found elsewhere [26, 27]. We excluded WHO members with missing vaccination rates, or GNI per capita; thus, our sample size in the merged dataset was 192 members. All datasets are publicly available and de-identified, therefore, a review from an Institutional Review Board was not required.

### Study variables

#### Primary outcome

The primary outcome of interest was *the cumulative number of persons fully vaccinated (i.e., receiving the required number of doses as per the vaccine guideline) per 100 population)*. Vaccination data were pooled from reports from members and WHO review of publicly available official data or data collected and published by third-party sites [28]. The population estimations for each country were extracted from the United Nations’ Department of Economic and Social Affairs, Eurostat, National Statistics Office Malta, The Government of the Pitcairn Islands, and Statistics Netherlands. A detailed description of the calculation can be found elsewhere [29, 30].

#### Secondary outcome

*Cumulative persons vaccinated with at least one dose per 100 population* was the secondary outcome of interest.

#### Exposure (s)

*GNI per capita* was calculated by the Word bank in U.S. dollars using the Atlas conversion factor. We created a categorical variable to classify members into different income levels based on the World Bank’s 2019 Gross National Income thresholds. GNI was classified into low income (less than $1,026), lower-middle income ($1,026 to $3,995), upper-middle income ($3,996-$12,375) and high income (more than $12,375). A detailed description of the calculation can be found elsewhere [26, 27]. *WHO regions* were our secondary exposure. WHO regions are classified into the Africa, Americas, South-East Asia, Europe, Eastern Mediterranean, and Western Pacific regions based on members respective WHO regional offices [4].

#### Covariates

The number of vaccine types in WHO members was the covariate. We include the number of vaccine types administered by WHO members as a covariate based on literature [20, 21] and a *priori* knowledge of possible confounding (availability of various vaccine choices). The number of COVID-19 vaccine types used by each WHO member was categorized into 1 to 3, 4 to 6, 7 to 9, and 10 to 12. We sourced the number of vaccine types data from the WHO data repository [28].

### Statistical analysis

We performed descriptive statistical analyses to determine the cumulative mean number of persons fully vaccinated per 100 population stratified by GNI per capita, WHO region, and the number of vaccine types used by WHO members. We performed ANOVA tests to assess significant differences in COVID-19 vaccination by WHO members’ GNI per capita, region, and the number of vaccine type used. We also performed negative binomial regression to assess the association between the vaccination rates and GNI per capital, WHO region and the number of vaccine types using two models. We used the negative binomial regression model because the COVID-19 vaccination outcomes were rates normalized from count data (i.e., only non-negative integer), and it managed for overdispersion in the dataset.

The first model looked at the crude association between vaccination rates and GNI per capital, WHO region and the number of vaccine types respectively. The second model examined the association between vaccination rates and GNI per capital adjusted for WHO region and the number of vaccine types. To adjust for differential follow-up time in the reporting of vaccination data between countries, we offset in the regression the natural log of the time (in weeks) between April 9^th^, 2021, and the most recent date each country member reported/updated vaccination data to WHO. April 9^th^, 2021, was the oldest “most recent” date a member reported vaccination data to WHO as of June 4^th^, 2022, therefore, was selected as the baseline follow-up date. We used SAS version 9.4 and STATA 16.1 to perform the analyses.

## Results

### Baseline characteristics

We examined a total of 192 WHO members. A description of the sample characteristics is provided in Tables 1 and 2. WHO members with high GNI per capita represented 33.85% of our sample and had the highest vaccination coverage [(73.92 ($\pm$ 12.89) based on the cumulative number of persons fully vaccinated and 77.44 ($\pm$ 13.93) based on the cumulative number of persons vaccinated with at least one dose per 100 population). The ANOVA test revealed statistically significant associations between GNI per capita and the aforementioned rates. Africa region had the lowest cumulative number of persons fully vaccinated per 100 population at 23.09 ($\pm$ 19.03), while countries in the Western Pacific region had the highest rate at 69.93 ($\pm$ 22.85) per 100. Similarly, members in the Africa region had the lowest cumulative persons vaccinated with at least one dose per 100 population at 28.75 ($\pm$ 20.26). While countries in the Western Pacific region had the highest cumulative persons vaccinated with at least one dose per 100 population at 28.75 ($\pm$ 20.26) at 76.03 ($\pm$ 23.31) per 100.

**Table 1:** Characteristics and differences in WHO members with population fully vaccinated

Variable	WHO members (Countries, areas, and territories)	Rate of Fully Vaccinated Residents per 100 population	*p*-value
	***N* = 192 (%)**	**M (± SD)**^**a, b**^	
**WHO Region (*n* = 6)**			< 0.0001
Africa	45 (23.44)	23.09 (19.03)	
Americas	39 (20.31)	59.98 (21.38)	
Eastern Mediterranean	20 (10.42)	45.34 (30.03)	
Europe	53 (27.60)	60.38 (17.96)	
South-East Asia	10 (5.21)	66.54 (11.34)	
Western Pacific	25 (13.02)	69.93 (22.85)	
**Vaccine Type**			0.0038
1 to 3	61 (31.77)	47.46 (29.87)	
4 to 6	88 (45.83)	55.24 (29.54)	
7 to 9	22 (11.46)	64.38 (17.19)	
10 to 12	21 (10.94)	46.21 (29.54)	
**GNI Per Capita**			< 0.0001
Low Income	23 (11.98)	15.90 (14.94)	
Lower Middle Income	48 (25.00)	40.30 (23.91)	
Upper Middle Income	56 (29.17)	49.89 (23.91)	
High Income	65 (33.85)	73.92 (12.89)	

*Abbreviations*: *SD* Standard deviation, *N* WHO Members (i.e., countries, areas, and territories), *n* Number of WHO regions, *M* Mean population, *%* percentage, Level of significance, *p* < 0.05

^a^mean cumulative number of persons fully vaccinated per 100 population

^b^WHO pooled COVID-19 vaccination data as of June 4, 2022

**Table 2:** Characteristics and differences in WHO members with population received at least one dose

	**WHO members** **(Countries, areas, and territories)**	**Rate persons vaccinated with at least one dose per 100 population**	***p*-value**
	***N* = 192 (%)**	**M (± SD)**^**a, b**^	
**WHO Region (*n* = 6)**			< 0.0001
Africa	45 (23.44)	28.75 (20.26)	
Americas	39 (20.31)	66.26 (22.50)	
Eastern Mediterranean	20 (10.42)	50.53 (29.21)	
Europe	53 (27.60)	63.27 (18.46)	
South-East Asia	10 (5.21)	74.45 (9.35)	
Western Pacific	25 (13.02)	76.03 (23.31)	
**Vaccine Type**			0.0063
1 to 3	61 (31.77)	49.59 (31.22)	
4 to 6	88 (45.83)	59.31 (23.73)	
7 to 9	22 (11.46)	71.09 (17.61)	
10 to 12	21 (10.94)	51.35 (28.72)	
**GNI per Capita**			< 0.0001
Low Income	23 (11.98)	20.17 (16.39)	
Lower Middle Income	48 (25.00)	47.73 (24.99)	
Upper Middle Income	56 (29.17)	55.33 (22.97)	
High Income	65 (33.85)	77.44 (13.93)	

*Abbreviations*: *SD* Standard deviation, *N* WHO Members (i.e., countries, areas, and territories); *n* Number of WHO regions, *M* Mean population, *%* percentage, Level of significance, *p* < 0.05

^a^mean cumulative number vaccinated with at least one dose; per 100 population

^b^WHO pooled COVID-19 vaccination data as of June 4, 2022

The majority of members reported administering 1 to 3 (31.77%) and 4 to 6 (45.83%) vaccine types (Table 1). The study also showed a statistically significant association between the number of vaccine-type administered and vaccination coverage (i.e., mean cumulative persons vaccinated with at least one dose and cumulative mean number of persons fully vaccinated per 100 population). Members offering 7 to 9 vaccine types had the highest mean cumulative persons vaccinated with at least one dose and cumulative mean number of persons fully vaccinated per 100 population at 71.09 ($\pm$ 17.61) and 64.38 ($\pm$ 17.19), respectively.

### Vaccination rate ratios

#### Cumulative number of persons fully vaccinated per 100 population

Using high GNI per capita as the reference, members with low GNI per capita had the lowest crude rate of persons fully vaccinated (RR 0.21, 95% CI 0.17–0.28). However, after adjusting for the number of vaccines used and the WHO region, the rate of persons fully vaccinated among low GNI per capita members was 0.30 times that of high GNI per capita members, as shown in Tables 3 and 4. Adjusting for the number of vaccine types used and GNI per capita, the rate of persons fully vaccinated and received at least one dose of the vaccine in the African region was 0.71 times (aRR 0.71, 95% CI 0.55–0.91) that of the European members.

**Table 3:** Association between GNI per capita, WHO regions, and unadjusted COVID-19 vaccination coverage

Variable	Rate Ratio of Cumulative number of persons fully vaccinated per 100 population among WHO Members	Rate Ratio of Cumulative persons vaccinated with at least one dose per 100 population among WHO Members
	**RR (95% CI) **^**a**^	**RR (95%CI) **^**b**^
**GNI Per Capita**		
Low Income	0.21 (0.17,0.28) ***	0.26 (0.20, 0.33) ***
Lower Middle Income	0.54 (0.45,0.66) ***	0.61 (0.52, 0.73) ***
Upper Middle Income	0.68 (0.57,0.81) ***	0.72 (0.61, 0.85) ***
High Income	REF	REF
**WHO Region**		
Europe	REF	REF
Africa	0.37 (0.30,0.47) ***	0.44 (0.36,0.54) ***
Americas	0.98 (0.78,1.23)	1.03 (0.84,1.26)
Eastern Mediterranean	0.72 (0.55,0.95) *	0.77 (0.59,0.99) *
South-East Asia	1.06 (0.74,1.51)	1.13 (0.81, 1.57)
Western Pacific	1.15 (0.89,1.48)	1.20 (0.95, 1.51)
**Vaccine Type**		
1 to 3	0.97 (0.71, 1.34)	1.00 (0.75, 1.34)
4 to 6	1.23 (0.91,1.68)	1.19 (0.90,1.58)
7 to 9	1.43 (0.97,2.10)	1.42 (0.99, 2.02)
10 to 12	REF	REF

*Abbreviations*: *95% CI* 95 percent Confidence Interval, *REF* Reference, *RR* Rate Ratio

^a^Crude Rate Ratio of Cumulative number of persons fully vaccinated per 100 population among WHO Members

^b^Crude Rate Ratio of Cumulative persons vaccinated with at least one dose per 100 population among WHO Members

^*^p < 0.05

^**^p < 0.01

^***^p < 0.001

**Table 4:** Association between GNI per capita, WHO regions, and adjusted COVID-19 vaccination coverage

Variable	Rate Ratio of Cumulative number of persons fully vaccinated per 100 population among WHO members	Rate Ratio of Cumulative number of vaccinated with at least one dose per 100 population among WHO members
	**aRR (95% CI) **^**a**^	**aRR (95%CI) **^**b**^
**GNI Per Capita**		
Low Income	0.30 (0.22, 0.40) ***	0.34 (0.26, 0.44) ***
Lower Middle Income	0.51 (0.42, 0.62) ***	0.57 (0.48, 0.69) ***
Upper Middle Income	0.61 (0.52, 0.72) ***	0.65 (0.55, 0.76) ***
High Income	REF	REF
**WHO Region**		
Europe	REF	REF
Africa	0.71 (0.55, 0.91) **	0.78 (0.62, 0.99) *
Americas	1.11 (0.92, 1.34)	1.15 (0.96, 1.37)
Eastern Mediterranean	0.74 (0.30, 1.81)	0.84 (0.36, 1.97)
South-East Asia	1.40 (1.01, 1.94) *	1.41 (1.04, 1.91) *
Western Pacific	1.40 (1.12,1.74) **	1.40 (1.14,1.73) **
**Vaccine Type**		
1 to 3	0.77 (0.32, 1.86)	0.87 (0.38, 1.98)
4 to 6	0.81 (0.34, 1.94)	0.88 (0.39, 2.02)
7 to 9	1.09 (0.45, 2.65)	1.16 (0.50, 2.69)
10 to 12	REF	REF

*Abbreviations*: *95% CI* 95 percent Confidence Interval, *REF* Reference, *aRR* Adjusted Rate Ratio

^a^Adjusted Rate Ratio of Cumulative number of persons fully vaccinated per 100 population (fully vaccinated rate = GNI + Region + type + ln(time))

^b^Adjusted Rate Ratio of Cumulative persons vaccinated with at least one dose per 100 population (vaccinated with at least 1 dose rate = GNI + Region + type + ln(time))

^*^p < 0.05

^**^p < 0.01

^***^p < 0.001

#### Cumulative persons vaccinated with at least one dose per 100 population

Members with low GNI per capita had the lowest adjusted rate of persons *with at least one* vaccination dose (aRR 0.34, 95% CI 0.26–0.44) compared to high GNI per capita members. The African region had a significantly lower rate of persons with at least one vaccination dose than European countries (aRR 0.78, 95% CI 0.62–0.99) adjusted for the number of vaccine types used and GNI per capita. Conversely, countries in the South-East Asia region were 1.41 times more likely to receive at least one dose of COVID-19 vaccines than countries in the European region, adjusted for the number of vaccine types administered and GNI per capita (95% CI 1.04—1.91). Similarly, countries in the Western Pacific region were 1.40 times more likely to receive at least one dose of COVID-19 vaccines than European countries, adjusted for the number of vaccine types administered (95% CI: 1.14–1.73).

## Discussion

We explored the association between WHO member’s GNI per capita, regions, and vaccination coverage. Lower-income members based on GNI per capita had significantly lower vaccination coverage. This finding confirms the existence of barriers regarding the affordability of vaccines as a major barrier to global vaccination efforts [5, 6]. This study also reveals the existence of disparities in vaccination despite efforts by COVAX and other global agencies to increase vaccine access [10, 11]. This finding adds to existing knowledge on vaccine hesitancy due to low education levels, and limited access to accurate health information [18, 31]. Thus, in addition to vaccination campaigns focusing on creating awareness about vaccine efficacy, safety, and benefits, promoting vaccine equity and affordability is warranted [32].

The study also revealed that African region had the lowest mean of persons per 100 population either fully or received at least one dose of the COVID-19 vaccine. A possible explanation for this would be the limited access, and availability of COVID-19 vaccines in this region [33], low perceptions toward vaccine effectiveness and the perceived risk of getting infected may also explain the discrepancies [34, 35]. Other potential reasons could be the inequitable distribution of vaccines in most African WHO members [5, 6, 36], attributable to poor health infrastructure, transportation, and cold-chain management challenges [23].

In contrast, the Western Pacific region reported significantly higher vaccination coverage for persons fully vaccinated and receiving with at least one dose of the vaccine. These high vaccination rates may be attributed to efforts by member countries to overcome vaccine access and hesitancy barriers in their region. For instance, Vietnam (which has fully vaccinated its population up to over 90%) developed strategies for reaching hard-to-reach people using mobile vaccination teams and home visits [37]. Similar outreach efforts have been employed in Malaysia (which has over 83% fully vaccination rates), where the ministry of health translated the vaccination information materials into more than 20 local languages to promote COVID-19 vaccination awareness and outreach [38].

This study contributes to a limited body of research on comparison of vaccination rates by WHO regions and member GNI per capita. Our findings underscore key disparities in vaccination rates, which highlight the need for enhanced support to address COVID-19 vaccine coverage globally. Other strengths of this study include the use of robust analytics (negative binomial regression) to assess associations between GNI per capita and the weighted cumulative number of persons vaccinated per 100 persons (population density) that controlled for the number of vaccine types administered by WHO members. Despite these strengths, specific limitations merit mention. First, we acknowledge the likelihood of ecological fallacy as findings from regions may not be inferred to individual levels. Secondly, we only analyzed data as of June 4, 2022, which is subject to change [28]. Thirdly, COVID-19 infection is dynamic, with WHO reporting new changes in reporting procedures, vaccine eligibility, and now booster doses. Therefore, continued monitoring of the situation and similar assessments are warranted. Relying on secondary data is another limitation which may contribute to inaccuracies in reporting (overreporting/underreporting), especially in countries and WHO members with poor healthcare systems. Finally, we acknowledge the impossibility of controlling for all confounders as we relied on the availability of data at the global level; thus, our study may contain residual confounding. Nonetheless, these findings would be beneficial in designing vaccination campaigns and strategies globally and in regions where vaccination coverage remains very low.

## Conclusion

GNI per capita was significantly associated with COVID-19 vaccination coverage. Our findings also suggest that lower income countries, territories, and areas, particularly those in Africa, had significantly lower vaccination coverage. Further attention should be given to these regions to address existing barriers to the COVID-19 vaccination coverage including income inequities. Future studies should explore the relationship between specific COVID-19 vaccine types and vaccination coverage at the global level.

## Data Availability

All data used in this analysis are de-identified and publicly available. COVID-19 vaccination data was sourced from the WHO COVID-19 data repository: https://covid19.who.int/info/ GNI per capita was retrieved from the World Bank’s 2019 Gross National Income data: https://data.worldbank.org/about. The population estimations for each country were extracted from the United Nations’ Department of Economic and Social Affairs, Eurostat, National statistics Office Malta, The Government of the Pitcairn Islands, and Statistics Netherlands (CBS): https://population.un.org/wpp/Download/Standard/Population

## Abbreviations

COVID-19: Coronavirus disease 2019
GNI: Gross National Income
OECD: Organization for Economic Cooperation and Development
SARS-CoV-2: Severe Acute Respiratory Syndrome Coronavirus-2
WHO: World Health Organization
COVAX: COVID-19 Vaccines Global Access
